# Supplementary material for: Characterization of Pseudomonas aeruginosa l,d-Transpeptidases and Evaluation of Their Role in Peptidoglycan Adaptation to Biofilm Growth
Source: Microbiol Spectr. 2023 May 31;11(4):e05217-22. doi: 10.1128/spectrum.05217-22 (PMC10434034; doi:10.1128/spectrum.05217-22)
Supplement: Supplemental file 1 — Supplemental material. Download spectrum.05217-22-s0001.pdf, PDF file, 1.2 MB [file spectrum.05217-22-s0001.pdf]

Supplementary Material

Characterization of *Pseudomonas aeruginosa* L,D-transpeptidases and evaluation of their role in peptidoglycan adaptation to biofilm growth

Inès Hugonneau-Beaufet[a], Jean-Philippe Barnier[a,b,c], Stanislas Thiriet-Rupert[d], Sylvie Létoffé[d], Jean-Luc Mainardi [a,b,c], Jean-Marc Ghigo[d], Christophe Beloin*[d], Michel Arthur*[a]

[a] Centre de Recherche des Cordeliers, Sorbonne Université, INSERM, Université Paris Cité, F-75006 Paris France

[b] Service de Microbiologie, Hôpital Européen Georges Pompidou, AP-HP Assistance Publique-Hôpitaux de Paris, F-75015 Paris France

[c] Université Paris Cité, Faculté de Santé, UFR de Médecine, F-75015 Paris France

[d] Institut Pasteur, Université Paris Cité, UMR CNRS 6047, Genetic of Biofilms laboratory, F-75015 Paris France

*Corresponding authors

Running title: ***Pseudomonas aeruginosa*** **L,D-transpeptidases**

Supplementary Table S1. Muropeptide profile of peptidolgycan extracted from a stationary phase culture of *P. aeruginosa* strain PA14

Fraction	RT (min)	Integration (mAU.ml)	Mass obs	Mass cal	ppm	Intensity	Structure
F3	22.96	6.1	518.232	NA	NA	5,600	Unkown
F4	22.96	6.1	870.371	870.371	-0.5	147,000	GMr-Tri
F5	24.41	1.6	976.383	976.386	2.1	22,000	GM-GMr (tetrasaccharide)
F6	25.33	2.4	738.328	738.328	0.2	69,000	Mr-Tetra
F9	27.86	27.6	941.406	941.408	2.1	215,000	GMr-Tetra
			998.463	998.466	2.6	18,000	GMr-Tri→K
F14	33.62	2.9	1154.566	1154.567	0.9	35,000	GMr-Tri→KR
			939.390	939.392	1.9	9,000	GM-Tetra
			1476.645	1476.645	0.6	1,000	GM-GMr-Tri→K
			1313.572	1313.572	0.2	1,000	Tri→GMr-Tetra
F16	35.4	1.5	679.301	NA	NA	18,000	Unknown
			1384.607	1384.609	1.5	19,000	GMr-Tetra→Tetra
F17	36.6	1.5	1722.724	1722.731	3.9	6,000	GMr-Tri→GMr-Tri
			1590.685	1590.687	0.8	9,000	GMr-Tri→Mr-Tetra
F18	37.38	3.6	1793.766	1793.768	1.0	45,000	GMr-Tetra→GMr-Tri (MS/MS)
F19	38.77	2.5	1793.764	1793.768	1.8	17,000	GMr-Tri→GMr-Tetra (MS/MS)
F20	39.47	27.2	1864.802	1864.805	1.5	208,000	GMr-Tetra→GMr-Tetra (MS/MS)
F25	44.34	2.1	905.388	NA	NA	6,000	Unknown
			2788.185	2788.202	6.1	15,000	GMr-Tetra→GMr-Tetra→GMr-Tetra
F27	46.56	14.4	1860.769	1860.774	2.2	8,000	GM-Tetra→GM-Tetra
			1773.737	1773.742	2.3	1,000	GMAnh-Tetra→GMr-Tri
			1038.445	NA	NA	6,000	Unknown
			3266.38	3266.382	0.6	100	GM-GMr-Tetra→GMr-Tetra→GMr-Tetra
F29	48.73	4.4	1844.774	1844.779	2.6	35,000	GMAnh-Tetra→GMr-Tetra

Fraction	*rp* HPLC fraction
RT (min)	Retention time
mAU.ml	Integration of the area under the abosorbance curve (λ=205 nm)
Mass obs	Observed monoistopic mass
Mass cal	Calculated monoistopic mass
ppm	Difference in part per million betwen the observed and calculated monoisotopic masses
Intensity	Current intensity for the indicated mass (arbitrary unit)
Structure	Structure of muropeptide. Abbreviations: GM, MurNAc-GlcNAc; Tri, tripeptide; Tetra, tetrapeptide; K and KR, OprI residues; In dimers, polymorphism in the disaccharide is arbitrarly indicated in the donor [r, reduced; anh, anhydro; Mr, absence of GlcNAc due to glucosaminidase activity; void, absence of GlcNAc and MurNAc due to amidase activity). MS/MS, the structure of the dimers was determined by tandem mass spectrometry.

Supplementary Table S2. Muropeptide profile of peptidoglycan extracted from *P. aeruginosa* PA14 grown in biofilm

Fraction	RT (min)	Integration (mAU.ml)	Mass obs	Mass cal	ppm	Intensity	Structure
F1	23.76	62.9	870.366	870.371	5.1	75,000	GMr-Tri
F2	25.24	23.4	976.381	976.386	4.8	152,000	GM-GMr (tetrasaccharide)
F4	28.65	128.1	941.404	941.408	3.7	920,000	GMr-Tetra
F5	29.18	14.7	998.462	998.466	3.5	435,000	GMr-Tri→K
F6	29.93	5.1	521.231	NA	NA	165,000	Unknown
F7	30.98	7.4	1348.546	1348.550	3.6	53,000	GM-GMr-Tri
F9	34.23	13.2	1154.565	1154.567	1.7	124,000	GMr-Tri→KR
F10	34.57	9.0	1419.579	1419.588	6.3	110,000	GM-GMr-Tetra
F11	35.79	16.7	1384.601	1384.609	5.9	230,000	GMr-Tetra→Tetra
F12	37.68	8.3	1793.757	1793.768	6.1	68,000	GMr-Tetra→GMr-Tri
F13	38.42	17.2	1793.765	1793.768	1.5	90,000	GMr-Tri→GMr-Tetra
F14	39.58	118.9	1864.800	1864.805	2.7	280,000	GMr-Tetra→GMr-Tetra
F17	42.23	9.2	2342.974	2342.985	4.4	56,000	GM-GMr-Tetra→GMr-Tetra
			2077.956	2077.964	3.6	10,000	GMr-Tetra→GMr-Tri→KR
F20	44.32	9.6	1844.767	1844.779	6.4	38,000	GMAnh-Tetra→GM-Tetra
			495.195	NA	NA	56,000	Unknown
F21	46.89	11.7	1773.736	1773.742	3.4	4,000	GMAnh-Tri→GMr-Tetra or GMAnh-Tetra→GMr-Tri
			1364.580	1364.583	2.6	28,000	GMAnh-Tetra→Tetra
F22	48.77	13.6	1844.776	1844.779	1.6	100,000	GMAnh-Tetra→GM-Tetra

Fraction	*rp* HPLC fraction
RT (min)	Retention time
mAU.ml	Integration of the area under the abosorbance curve (λ=205 nm)
Mass obs	Observed monoistopic mass
Mass cal	Calculated monoistopic mass
ppm	Difference in part per million betwen the observed and calculated monoisotopic masses
Intensity	Current intensity for the indicated mass (arbitrary unit)
Structure	Structure of muropeptide. Abbreviations: GM, MurNAc-GlcNAc; Tri, tripeptide; Tetra, tetrapeptide; K and KR, OprI residues; In dimers, polymorphism in the disaccharide is arbitrarly indicated in the donor [r, reduced; anh, anhydro; void, absence of GlcNAc and MurNAc due to amidase activity).

Supplementary Table S3. Antibiotic susceptibility testing by disk diffusion assay

	Inhibition zones (mm) for derivatives of strains of *P. aeruginosa* PA14				
**Disk (loading dose)**	**WT**	**Δ*ldt*** $_{Pae1}$	**Δ*ldt*** $_{Pae2}$<br>**Δ*ldt*** $_{Pae3}$	**Δ*ldt*** $_{Pae1}$<br>**Δ*ldt*** $_{Pae2}$<br>**Δ*ldt*** $_{Pae3}$	**Δ*oprI***
Ampicillin (10 µg)	<6	<6	<6	<6	<6
Amoxicillin (20 µg)/Clavulanic Acid (10 µg)	<6	<6	<6	<6	<6
Ticarcillin (75 µg)	26	25	25	25	26
Piperacillin (30 µg)	29	28	27	27	28
Piperacillin (30 µg)/Tazobactam (6 µg)	26	26	26	26	25
Colistin (30 µg)	<6	<6	<6	<6	<6
Cefmandole (30 µg)	<6	<6	<6	<6	<6
Cefoxitin (30 µg)	<6	<6	<6	<6	<6
Cefixime (5 µg)	<6	<6	<6	<6	<6
Cefotaxime (5 µg)	<6	<6	<6	<6	<6
Ceftazidime (10 µg)	27	27	27	26	26
Cefepime (30 µg)	34	33	32	32	31
Aztreonam (30 µg)	29	29	29	29	29
Vancomycin (5 µg)	<6	<6	<6	<6	<6
Ertapenem (10 µg)	24	24	24	25	23
Imipenem (10 µg)	34	33	34	34	34
Meropenem (10 µg)	40	42	39	42	40
Mecillinam (10 µg)	<6	<6	<6	<6	<6
Ticarcillin (75 µg)/Clavulanic Acid (10 µg)	26	26	25	26	26
Temocillin (30 µg)	<6	<6	<6	<6	<6
Tobramycin (10 µg)	28	28	27	27	28
Netilmicin (10 µg)	20	18	17	15	20
Amikacin (30 µg)	29	28	27	26	28
Nalidixic Acid (30 µg)	12	11	12	11	10
Ciprofloxacin (5 µg)	42	41	41	42	41
Norfloxacin (10 µg)	38	37	37	38	37
Erythromycin (15 µg)	11	10	11	10	12
Trimethoprime (1.25 µg)/Sulphamethoxazole (23.75 µg)	9	<6	10	<6	9
Linezolide (10 µg)	<6	<6	<6	<6	<6
Fosfomycin (200 µg)	26	25	27	25	24
Colistin (50 µg)	20	20	20	20	19

Values are the medians of three biological repeats.

<6 when bacteria grew at the contact of the disks.

26

Supplementary Table S4. Impact of *ldt* and *oprI* deletions on the swimming motility of *P. aeruginosa* PA14

**Deletion**	**Swimming diameter (mm)**
None	23
Δldt_{Pae1}	23
$\Delta ldt_{Pae2} \Delta ldt_{Pae3}$	25
$\Delta ldt_{Pae1} \Delta ldt_{Pae2} \Delta ldt_{Pae3}$	25
Δ*oprI*	23

Values are the medians of three biological repeats.

	E. coli _YcbB	Sty_YcbB	E. coli _YafK	Sty_YafK	Ldt$_{Pae3}$	Ldt$_{Pae2}$	E. coli _ErfK	Sty_ErfK	E. coli _YbiS	Sty_Ybis	Cje_YbiS	E. coli _YnhG	Sty_YnhG
E. coli _YcbB	100,00	92,22	11,65	11,65	14,29	24,19	21,14	21,14	21,14	21,14	21,95	23,58	22,76
Sty_YcbB	92,22	100,00	10,58	10,58	12,50	23,20	20,97	20,97	19,51	19,51	20,97	23,39	22,58
E. coli _YafK	11,65	10,58	100,00	93,13	30,63	16,67	19,47	19,47	22,02	22,02	17,24	18,42	18,42
Sty_YafK	11,65	10,58	93,13	100,00	29,73	15,79	18,58	18,58	21,10	21,10	18,10	18,42	18,42
Ldt$_{Pae3}$	14,29	12,50	30,63	29,73	100,00	20,63	19,84	19,84	17,89	17,89	15,87	24,41	25,20
Ldt$_{Pae2}$	24,19	23,20	16,67	15,79	20,63	100,00	46,27	48,51	52,31	51,54	48,51	47,73	47,73
E. coli _ErfK	21,14	20,97	19,47	18,58	19,84	46,27	100,00	88,89	74,81	75,57	72,59	53,79	51,52
Sty_ErfK	21,14	20,97	19,47	18,58	19,84	48,51	88,89	100,00	76,34	76,34	73,33	52,27	50,76
E. coli _YbiS	21,14	19,51	22,02	21,10	17,89	52,31	74,81	76,34	100,00	97,71	84,73	52,71	50,39
Sty_Ybis	21,14	19,51	22,02	21,10	17,89	51,54	75,57	76,34	97,71	100,00	84,73	53,49	51,16
Cje_YbiS	21,95	20,97	17,24	18,10	15,87	48,51	72,59	73,33	84,73	84,73	100,00	50,76	49,24
E. coli _YnhG	23,58	23,39	18,42	18,42	24,41	47,73	53,79	52,27	52,71	53,49	50,76	100,00	94,12
Sty_YnhG	22,76	22,58	18,42	18,42	25,20	47,73	51,52	50,76	50,39	51,16	49,24	94,12	100,00
E. coli _YcfS	20,80	20,63	21,55	22,41	20,93	48,51	62,69	61,94	63,36	63,36	61,94	61,03	58,82
Sty_ycfS	21,60	20,63	21,55	22,41	20,16	49,25	61,19	60,45	62,60	61,83	61,19	59,56	59,56
Cje_Ycfs	21,95	21,77	18,64	18,64	21,26	48,87	64,66	66,92	62,02	62,02	59,56	64,71	62,50
Ldt$_{Nme}$	23,97	22,95	15,93	15,93	18,70	36,15	25,58	27,13	29,60	29,60	27,27	34,11	35,66
Bs_YkuD	23,71	22,45	21,74	20,65	27,18	32,71	35,24	35,24	33,66	33,66	29,91	35,58	33,65
Ldt$_{Cd2}$	23,64	22,73	20,39	19,42	23,89	31,93	36,75	35,90	34,21	35,09	30,77	34,19	33,33
Bs_Yqjb	18,18	17,27	19,42	19,42	26,55	31,93	36,75	35,90	32,46	34,21	28,21	31,62	30,77
Ldt$_{Mt6}$	18,90	17,97	12,00	13,00	14,55	15,89	11,32	14,15	10,78	10,78	13,21	12,38	13,33
Ldt$_{Mab6}$	16,24	16,24	9,41	10,59	11,34	15,22	13,04	14,13	13,04	13,04	15,22	11,96	11,96
Ldt$_{fs}$	18,52	16,67	13,98	12,90	18,27	24,24	16,00	17,00	15,31	15,31	16,00	16,16	16,16
Ldt$_{Cd1}$	21,98	20,88	14,29	15,58	20,45	15,66	14,29	14,29	18,07	19,28	19,05	21,69	21,69
Ldt$_{fm}$	16,50	16,35	15,96	17,02	19,61	25,25	27,27	26,26	26,32	26,32	24,00	30,61	28,57
Ldt$_{Pae11}$	10,23	9,09	9,89	9,89	20,83	21,51	15,05	16,13	15,22	15,22	16,13	16,13	18,28
Ldt$_{Cbu2}$	18,37	19,19	10,75	10,75	15,00	22,00	16,00	16,00	16,49	16,49	15,00	18,37	19,39
Bs_YciB	8,65	8,57	9,68	9,68	12,12	18,56	23,47	22,45	26,60	26,60	25,25	19,79	18,75
Ldt$_{Pae1}$	15,73	16,67	12,36	12,36	17,02	29,03	23,91	25,00	26,14	25,00	27,96	25,27	27,47
Ldt$_{Cd3}$	14,71	14,56	13,19	13,19	13,13	16,33	18,37	17,35	19,15	19,15	20,41	14,58	16,67
Ldt$_{Mt5}$	7,55	6,54	9,18	9,18	15,60	17,59	19,63	19,63	17,48	16,50	15,89	18,87	17,92
Ldt$_{Mab5}$	10,38	9,35	11,34	11,34	14,68	18,69	19,63	20,56	19,42	19,42	16,82	19,81	19,81
Ldt$_{Mt3}$	15,15	15,15	14,81	14,81	12,77	15,38	21,98	21,98	22,47	21,35	24,18	23,08	21,98
Ldt$_{Mab3}$	14,29	14,29	15,07	15,07	12,64	18,07	24,10	21,69	23,46	24,69	25,30	24,10	22,89
Ldt$_{Mt1}$	15,32	14,29	13,68	14,74	16,82	17,14	17,14	19,05	16,83	16,83	18,10	18,27	18,27
Ldt$_{Mab1}$	13,46	14,42	12,79	12,79	17,17	17,71	18,75	21,88	17,02	18,09	21,88	18,75	18,75
Ldt$_{Mt2}$	13,51	13,39	10,42	11,46	17,76	17,92	17,14	19,05	18,81	18,81	20,95	16,35	15,38
Ldt$_{Mab2}$	16,51	16,51	9,89	9,89	16,35	20,79	14,85	14,85	17,17	18,18	19,80	16,83	16,83
Ldt$_{Mt4}$	9,82	9,73	9,28	9,28	14,81	14,95	17,92	17,92	18,63	19,61	21,70	19,05	18,10
Ldt$_{Mab4}$	16,22	16,22	10,75	10,75	17,92	20,39	17,48	19,42	17,82	18,81	17,48	22,33	22,33

	E. coli _YcfS	Sty_ycfS	Cje_Ycfs	Ldt$_{Nme}$	Bs_YkuD	Ldt$_{Cd2}$	Bs_Yqjb	Ldt$_{Mt6}$	Ldt$_{Mab6}$	Ldt$_{fs}$	Ldt$_{Cd1}$	Ldt$_{fm}$	Ldt$_{Pae11}$
E. coli _YcbB	20,80	21,60	21,95	23,97	23,71	23,64	18,18	18,90	16,24	18,52	21,98	16,50	10,23
Sty_YcbB	20,63	20,63	21,77	22,95	22,45	22,73	17,27	17,97	16,24	16,67	20,88	16,35	9,09
E. coli _YafK	21,55	21,55	18,64	15,93	21,74	20,39	19,42	12,00	9,41	13,98	14,29	15,96	9,89
Sty_YafK	22,41	22,41	18,64	15,93	20,65	19,42	19,42	13,00	10,59	12,90	15,58	17,02	9,89
Ldt$_{Pae3}$	20,93	20,16	21,26	18,70	27,18	23,89	26,55	14,55	11,34	18,27	20,45	19,61	20,83
Ldt$_{Pae2}$	48,51	49,25	48,87	36,15	32,71	31,93	31,93	15,89	15,22	24,24	15,66	25,25	21,51
E. coli _ErfK	62,69	61,19	64,66	25,58	35,24	36,75	36,75	11,32	13,04	16,00	14,29	27,27	15,05
Sty_ErfK	61,94	60,45	66,92	27,13	35,24	35,90	35,90	14,15	14,13	17,00	14,29	26,26	16,13
E. coli _YbiS	63,36	62,60	62,02	29,60	33,66	34,21	32,46	10,78	13,04	15,31	18,07	26,32	15,22
Sty_Ybis	63,36	61,83	62,02	29,60	33,66	35,09	34,21	10,78	13,04	15,31	19,28	26,32	15,22
Cje_YbiS	61,94	61,19	59,56	27,27	29,91	30,77	28,21	13,21	15,22	16,00	19,05	24,00	16,13
E. coli _YnhG	61,03	59,56	64,71	34,11	35,58	34,19	31,62	12,38	11,96	16,16	21,69	30,61	16,13
Sty_YnhG	58,82	59,56	62,50	35,66	33,65	33,33	30,77	13,33	11,96	16,16	21,69	28,57	18,28
E. coli _YcfS	100,00	93,48	69,85	29,01	32,08	33,61	31,09	14,95	14,89	17,82	18,82	27,27	18,95
Sty_ycfS	93,48	100,00	69,85	29,77	32,08	33,61	30,25	16,82	15,96	19,80	17,65	26,26	18,95
Cje_Ycfs	69,85	69,85	100,00	30,08	33,64	33,05	33,90	13,21	13,04	18,18	20,48	30,00	19,35
Ldt$_{Nme}$	29,01	29,77	30,08	100,00	35,78	28,33	32,50	13,73	11,36	20,20	24,10	21,21	17,20
Bs_YkuD	32,08	32,08	33,64	35,78	100,00	45,79	45,79	8,70	9,09	20,88	17,28	26,88	14,12
Ldt$_{Cd2}$	33,61	33,61	33,05	28,33	45,79	100,00	51,61	9,09	11,76	22,22	20,48	25,51	12,90
Bs_Yqjb	31,09	30,25	33,90	32,50	45,79	51,61	100,00	12,12	12,94	21,21	20,48	26,53	15,05
Ldt$_{Mt6}$	14,95	16,82	13,21	13,73	8,70	9,09	12,12	100,00	68,35	17,09	22,58	17,12	16,30
Ldt$_{Mab6}$	14,89	15,96	13,04	11,36	9,09	11,76	12,94	68,35	100,00	14,29	25,27	17,89	14,81
Ldt$_{fs}$	17,82	19,80	18,18	20,20	20,88	22,22	21,21	17,09	14,29	100,00	39,25	38,02	17,89
Ldt$_{Cd1}$	18,82	17,65	20,48	24,10	17,28	20,48	20,48	22,58	25,27	39,25	100,00	40,00	12,50
Ldt$_{fm}$	27,27	26,26	30,00	21,21	26,88	25,51	26,53	17,12	17,89	38,02	40,00	100,00	18,28
Ldt$_{Pae11}$	18,95	18,95	19,35	17,20	14,12	12,90	15,05	16,30	14,81	17,89	12,50	18,28	100,00
Ldt$_{Cbu2}$	16,00	18,00	18,18	16,67	16,67	15,62	15,62	12,39	13,83	13,98	19,23	20,21	16,30
Bs_YciB	23,47	22,45	20,41	12,63	18,89	15,05	16,13	13,04	15,69	19,27	21,98	20,75	17,05
Ldt$_{Pae1}$	30,11	31,18	31,18	23,33	18,89	19,10	19,10	18,27	22,47	24,18	25,00	26,97	30,95
Ldt$_{Cd3}$	17,35	17,35	16,49	13,40	24,18	20,62	18,56	10,38	12,09	17,59	23,66	16,36	13,83
Ldt$_{Mt5}$	15,74	16,67	19,63	21,50	14,00	12,15	18,69	16,07	15,46	16,67	17,39	18,52	14,14
Ldt$_{Mab5}$	17,59	20,37	20,56	21,50	14,14	14,15	19,81	13,51	13,40	19,44	16,30	22,43	15,15
Ldt$_{Mt3}$	21,51	21,51	23,08	20,88	18,07	18,68	17,58	13,40	16,84	21,00	26,88	27,55	14,94
Ldt$_{Mab3}$	22,35	22,35	28,92	22,89	18,67	19,28	21,69	16,48	20,22	20,65	29,41	31,11	16,46
Ldt$_{Mt1}$	19,81	17,92	20,00	17,14	24,74	17,31	17,31	17,12	16,49	16,07	27,08	27,68	21,21
Ldt$_{Mab1}$	21,43	20,41	22,92	18,75	23,86	16,67	19,79	15,69	15,46	23,81	28,12	29,13	20,65
Ldt$_{Mt2}$	15,09	14,15	17,14	17,14	21,43	20,95	15,24	14,29	16,49	16,07	26,04	25,66	12,12
Ldt$_{Mab2}$	12,62	12,62	14,85	17,82	21,51	16,83	11,88	14,95	17,53	20,00	28,12	25,00	13,40
Ldt$_{Mt4}$	17,76	15,89	16,98	17,92	16,16	15,09	13,21	17,54	19,19	19,47	31,96	26,55	18,18
Ldt$_{Mab4}$	21,90	20,00	23,30	18,45	22,11	19,42	19,42	15,45	16,16	20,54	28,87	29,36	17,35

	Ldt$_{Cbu2}$	Bs_YciB	Ldt$_{Pae1}$	Ldt$_{Cd3}$	Ldt$_{Mt5}$	Ldt$_{Mab5}$	Ldt$_{Mt3}$	Ldt$_{Mab3}$	Ldt$_{Mt1}$	Ldt$_{Mab1}$	Ldt$_{Mt2}$	Ldt$_{Mab2}$	Ldt$_{Mt4}$	Ldt$_{Mab4}$
E. coli _YcbB	18,37	8,65	15,73	14,71	7,55	10,38	15,15	14,29	15,32	13,46	13,51	16,51	9,82	16,22
Sty_YcbB	19,19	8,57	16,67	14,56	6,54	9,35	15,15	14,29	14,29	14,42	13,39	16,51	9,73	16,22
E. coli _YafK	10,75	9,68	12,36	13,19	9,18	11,34	14,81	15,07	13,68	12,79	10,42	9,89	9,28	10,75
Sty_YafK	10,75	9,68	12,36	13,19	9,18	11,34	14,81	15,07	14,74	12,79	11,46	9,89	9,28	10,75
Ldt$_{Pae3}$	15,00	12,12	17,02	13,13	15,60	14,68	12,77	12,64	16,82	17,17	17,76	16,35	14,81	17,92
Ldt$_{Pae2}$	22,00	18,56	29,03	16,33	17,59	18,69	15,38	18,07	17,14	17,71	17,92	20,79	14,95	20,39
E. coli _ErfK	16,00	23,47	23,91	18,37	19,63	19,63	21,98	24,10	17,14	18,75	17,14	14,85	17,92	17,48
Sty_ErfK	16,00	22,45	25,00	17,35	19,63	20,56	21,98	21,69	19,05	21,88	19,05	14,85	17,92	19,42
E. coli _YbiS	16,49	26,60	26,14	19,15	17,48	19,42	22,47	23,46	16,83	17,02	18,81	17,17	18,63	17,82
Sty_Ybis	16,49	26,60	25,00	19,15	16,50	19,42	21,35	24,69	16,83	18,09	18,81	18,18	19,61	18,81
Cje_YbiS	15,00	25,25	27,96	20,41	15,89	16,82	24,18	25,30	18,10	21,88	20,95	19,80	21,70	17,48
E. coli _YnhG	18,37	19,79	25,27	14,58	18,87	19,81	23,08	24,10	18,27	18,75	16,35	16,83	19,05	22,33
Sty_YnhG	19,39	18,75	27,47	16,67	17,92	19,81	21,98	22,89	18,27	18,75	15,38	16,83	18,10	22,33
E. coli _YcfS	16,00	23,47	30,11	17,35	15,74	17,59	21,51	22,35	19,81	21,43	15,09	12,62	17,76	21,90
Sty_ycfS	18,00	22,45	31,18	17,35	16,67	20,37	21,51	22,35	17,92	20,41	14,15	12,62	15,89	20,00
Cje_Ycfs	18,18	20,41	31,18	16,49	19,63	20,56	23,08	28,92	20,00	22,92	17,14	14,85	16,98	23,30
Ldt$_{Nme}$	16,67	12,63	23,33	13,40	21,50	21,50	20,88	22,89	17,14	18,75	17,14	17,82	17,92	18,45
Bs_YkuD	16,67	18,89	18,89	24,18	14,00	14,14	18,07	18,67	24,74	23,86	21,43	21,51	16,16	22,11
Ldt$_{Cd2}$	15,62	15,05	19,10	20,62	12,15	14,15	18,68	19,28	17,31	16,67	20,95	16,83	15,09	19,42
Bs_Yqjb	15,62	16,13	19,10	18,56	18,69	19,81	17,58	21,69	17,31	19,79	15,24	11,88	13,21	19,42
Ldt$_{Mt6}$	12,39	13,04	18,27	10,38	16,07	13,51	13,40	16,48	17,12	15,69	14,29	14,95	17,54	15,45
Ldt$_{Mab6}$	13,83	15,69	22,47	12,09	15,46	13,40	16,84	20,22	16,49	15,46	16,49	17,53	19,19	16,16
Ldt$_{fs}$	13,98	19,27	24,18	17,59	16,67	19,44	21,00	20,65	16,07	23,81	16,07	20,00	19,47	20,54
Ldt$_{Cd1}$	19,23	21,98	25,00	23,66	17,39	16,30	26,88	29,41	27,08	28,12	26,04	28,12	31,96	28,87
Ldt$_{fm}$	20,21	20,75	26,97	16,36	18,52	22,43	27,55	31,11	27,68	29,13	25,66	25,00	26,55	29,36
Ldt$_{Pae11}$	16,30	17,05	30,95	13,83	14,14	15,15	14,94	16,46	21,21	20,65	12,12	13,40	18,18	17,35
Ldt$_{Cbu2}$	100,00	17,35	30,53	20,41	19,61	18,81	28,74	30,86	29,70	30,43	21,57	21,65	25,49	21,43
Bs_YciB	17,35	100,00	35,58	31,63	14,42	15,38	22,22	25,61	21,36	23,16	18,45	24,24	23,81	19,61
Ldt$_{Pae1}$	30,53	35,58	100,00	25,00	18,95	18,09	29,49	32,86	28,57	30,12	17,39	29,89	27,66	25,56
Ldt$_{Cd3}$	20,41	31,63	25,00	100,00	20,00	17,43	29,41	27,66	23,28	25,23	21,37	23,21	20,51	20,35
Ldt$_{Mt5}$	19,61	14,42	18,95	20,00	100,00	75,21	34,62	35,42	32,20	38,53	33,61	30,70	37,19	35,90
Ldt$_{Mab5}$	18,81	15,38	18,09	17,43	75,21	100,00	32,69	33,33	29,66	33,03	29,66	27,19	34,17	31,62
Ldt$_{Mt3}$	28,74	22,22	29,49	29,41	34,62	32,69	100,00	73,79	52,25	57,66	46,85	42,34	45,95	40,54
Ldt$_{Mab3}$	30,86	25,61	32,86	27,66	35,42	33,33	73,79	100,00	52,43	53,40	44,66	41,75	54,37	46,60
Ldt$_{Mt1}$	29,70	21,36	28,57	23,28	32,20	29,66	52,25	52,43	100,00	69,83	47,20	41,32	52,00	46,72
Ldt$_{Mab1}$	30,43	23,16	30,12	25,23	38,53	33,03	57,66	53,40	69,83	100,00	45,69	43,97	52,59	53,45
Ldt$_{Mt2}$	21,57	18,45	17,39	21,37	33,61	29,66	46,85	44,66	47,20	45,69	100,00	69,42	53,97	56,56
Ldt$_{Mab2}$	21,65	24,24	29,89	23,21	30,70	27,19	42,34	41,75	41,32	43,97	69,42	100,00	52,07	57,02
Ldt$_{Mt4}$	25,49	23,81	27,66	20,51	37,19	34,17	45,95	54,37	52,00	52,59	53,97	52,07	100,00	62,90
Ldt$_{Mab4}$	21,43	19,61	25,56	20,35	35,90	31,62	40,54	46,60	46,72	53,45	56,56	57,02	62,90	100,00

Supplementary Table S5. Heat map of sequence identity in YkuD catalytic domains of LDTs. The percent identity was deduced from the Clustal Omega alignment shown in Supplementary Fig. S2. Color code for functions: green, 3→3 cross-linking; orange, lipoprotein or β-barrel protein anchoring; purple, hydrolysis of the tripeptide→lipoprotein amide bond; black, no experimental evidence available.

Supplementary Table S6. Characteristics and origin of the plasmids used in this study.

Plasmid	Characteristics	Origin
Vectors		
pHV6	Tet^R P_{trc} *lacI* *oriV* CloDF13	(53)
pET-TEV	Km^R P_{T7} *lacI* *oriV* ColE1	(61)
Recombinant plasmids for heterospecific expression of ldt_{Pae1}, ldt_{Pae2}, *and* ldt_{Pae3}		
pIHB1	pHV6Ωldt_{Pae1}	This study
pIHB2	pHV6Ωldt_{Pae2}	This study
pIHB3	pHV6Ωldt_{Pae3}	This study
Recombinant plasmid for protein production		
pET-TEVΩldt_{Pae1}	Production of soluble Ldt_{Pae1}	This study
Recombinant plasmids for ycbB and relA' expression		
pKT8	pHV7Ω*relA'*	(7)
pHV63	pHV7Ω*ycbB*	(53)

Supplementary Table S7. Characteristics and origin of *P. aeruginosa* strains used in this study.

Strain	Characteristics	Origin
E. coli BW25113	Δ(*araD-araB*)*567* Δ(*rhaD-rhaB*)*568* Δ*lacZ4787* (::rrnB-3) *hsdR514 rph-1*	(62)
E. coli BW25113Δ*6ldt*	Δ*ycbB* Δ*ynhG* Δ*ybiS* Δ*erfK* Δ*ycfS* Δ*yafK*	(26)
E. coli BW25113Δ*relA*	Δ*relA* derivative of BW25113	(7)
P. aeruginosa PA14	Wild-type strain	(63)
P. aeruginosa PA14Δldt_{Pae1}	Deletion of ldt_{Pae1} by MAR2xT7 transposon insertion, gentamicin^{R}	(64)
P. aeruginosa PA14Δldt_{Pae2}	Deletion of ldt_{Pae2} by MAR2xT7 transposon insertion, gentamicin^{R}	(64)
P. aeruginosa PA14Δldt_{Pae3}	Deletion of ldt_{Pae3} by MAR2xT7 transposon insertion, gentamicin^{R}	(64)
P. aeruginosa PA14Δldt_{Pae2}Δldt_{Pae3}	Deletion of ldt_{Pae2} by two-step allelic exchange in the PA14Δldt_{Pae3} strain	This study
P. aeruginosa PA14Δldt_{Pae1}Δldt_{Pae2}Δldt_{Pae3}	Deletion of ldt_{Pae1} by two-step allelic exchange in the PA14Δldt_{Pae2} Δldt_{Pae3} strain	This study
P. aeruginosa PA14Δ*oprI*	Deletion of *oprI* by two-step allelic exchange	This study

```
E. coli Lpp         MKAT-KLVLGAVILGSTLLAGCSSNAKIDQ--LS--SDVQTLN-AKVDQLSNDVNAMRSDVQAAKDDAARANQRLDNMATKY-RK
                    |    |    |  |      ||||  |     |    |      |  |             | |   |  || |   |  |  ||
P. aeruginosa OprI  MNNVLKFS--ALALAAVLATGCSSHSKETEARLTATEDAAARAQARADEAYRKADEALGAAQKAQQTADEANERALRMLEKASRK
```

Supplementary Figure S1. Alignment of the sequences of *E. coli* Lpp and *P. aeruginosa* OprI lipoproteins. The conserved GCSS motif and Arg-Lys C-terminus are highlighted in yellow and green, respectively.

```
E.coli_YcbB      ---SEAIYLHDTPNHNLF-------KRDTRALSSGCVRVNKASDLA--NMLLQ-------     167
Sty_ycbB         ---SDAIYLHDTPNHTLF-------QRDARALSSGCVRVNKASELA--NMLLQ-------     168
E.coli_YafK      GYEGKYLMIH------------------GDCVSIGCYAMTNQGIDEIFQFVTGALVFGQP     125
Sty_YafK         GYDGKYLMIH------------------GACVSVGCYAMTDSGIDEIFQFVTAALVFGQP     125
LdtPae3          --AGGMIMIHGTPLDDEYPE----WYFSTLDWTNGCIAMNNTDMREVWSVVKDGTLIEI-     135
LdtPae2          ---VPGYLIHGSNKKFGI----------GTRTSHGCFRMYNADVTHLFSMISVGTSVRI-     136
E.coli_ErfK      ---GRLYAIHGTNANFGI----------GLRVSQGCIRLRNDDIKYLFDNVPVGTRVQI-     135
Sty_erfK         ---GRLYAIHGTNANFGI----------GLRVSQGCIRLRNDDIKYLFDHVPVGTRVQI-     135
E.coli_YbiS      ---GRLYAIHGTNANFGI----------GLRVSHGCVRLRNEDIKFLFEKVPVGTRV---     131
Sty_Ybis         ---GRLYAIHGTNANFGI----------GLRVSHGCVRLRNDDIKFLFENVPVGTRV---     131
Cje_YbiS         ---GRLYAVHGTNANFGI----------GLRVSHGCVRLRDADIKWLYDNVPQGTRVQFI     136
E.coli_YnhG      --GNGEYLIHGTSAPDSV----------GLRVSSGCIRMNAPDIKALFSSVRTGTPVKV-     136
Sty_YnhG         --GNGEYLIHGTSAPDSV----------GLRVSSGCIRMNAPDIKALFAQVRTGTPVKV-     136
E.coli_YcfS      --YGGVYLLHGTNADFGI----------GMRVSSGCIRLRDDDIKTLFSQVTPGTKVNI-     138
Sty_ycfS         --YGGVYLLHGTNADFGI----------GMRVSSGCIRLRDGDIETLFRQVTPGTKVNI-     138
Cje_ycfs         --GTGQYLIHGTNANFGI----------GMRVSSGCIRLRPDDIEALFNSVPKGTRVQII     138
LdNme            --PKLGLGIHGTNAPASV----------PGVRSHGCVRMKSPDALEFAKTIASGSPASVI     133
Bs_YkuD          ----QHYGIHGTNNPASI----------GKAVSKGCIRMHNKDVIELASIVPNGTRVTIN     109
Ldtcd2           GTYGDTYGIHGNNNESSI----------GKHISGGCIRMHNKDVRWLFDQVPVGSDVII-     124
Bs_Yqjb          GTDGRIYGIHGTNREESV----------GKFVSNGCIRMHNDEVVHLFQTIPVGTRVLI-     124
LdtMt6           ---VMGVNK----AKVPGKGSAFFFHTTDGGPTAGCVAIDDATLVQIIRWLRPGAVIAI-     178
LdtMab6          ---VMGVNK----DRVPGGGSAFFVHSTDGGPTAGCVSLDDATLVKLIGWLRPGAVI---     158
Ldtfs            ---VTQIGIHDSDHKLDKYGDKEAFK--TDAGSNGCINTPGTEVSKIFDVSYDGMPVII-     131
Ldtcd1           ----GNIGIHDATWRNSFGGS---IY--KSSGSHGCVNTPFSKAKKIYENIEPGTPII--     107
Ldtfm            ----SGVGIHDSDWQPEYGGD---LW--KTRGSHGCINTPPSVMKELFGMVEKGTPVLVF     126
LdtPae11         ---WSVVQVSN-P---GQGL--------TPSEQLGKIRMDPTFVREMLGAMDVGSTLV--     99
LdtCbu2          ---SGGNAIHGSPYI----S--------NRNTSHGCIRVYPGAAAWLSHYFMRAGTKV--     132
Bs_YciB          -------IKTE-----AEKL--------GTKASHGCIRLTIPDAKWVYENIPEHTKVVIS     126
LdtPae1          ---WDGIALHA-----GNLP--------GYPASHGCIRLPMAFAKKLYGITGFSSTTVII     111
Ldtcd3           ------LYDSTGSYIIDGRL--------GEALSHGCIRLSTENAKWIYDNIPDTTTVII-     120
LdtMt5           ---NNGEFIHANPMSAGAQG--------NSNVTNGCINLSTENAEQYYRSAVYGDPVEV-     122
LdtMab5          ---NNGEFIHANPNTIGQQG--------NTNVTNGCINLSLGDAESYFRTAIYGDPVEV-     121
LdtMt3           ---SRGLYVHSAPWALPALG--------LENVSHGCISLSREDAEWYYNAVDIGDPVIV-     111
LdtMab3          ---TRGLFVHSAPWAVPAMG--------YENVSHGCISLPPAAAEWYFNNVNIGDPVVV-     103
LdtMt1           ---WSGVYVHSAPWSVNSQG--------YANVSHGCINLSPDNAAWYFDAVTVGDPIEV-     125
LdtMab1          ---WGGVYVHSAPWSTGAQG--------NSNVSHGCINLSPDNASWYYNTVSIGDPIII-     116
LdtMt2           ---YSGVFVHSAPWSVGAQG--------HTNTSHGCLNVSPSNAQWFYDHVKRGDIVEV-     126
LdtMab2          ---YSGIYVHAAPWSVGAQG--------RTNTSHGCLNVSTANAKWFYENTKRGDVVIV-     121
LdtMt4           ---NSGNFVHSAPWSVADQG--------KRNVTHGCINLSPANAKWFYDNFGSGDPVVV-     128
LdtMab4          ---NSGIFVHAAPWSVGAQG--------KSDTSHGCINVNTDNATWFFNQSHPGDPVIV-     124
```

Supplementary Figure S2. Multiple sequence alignment of a portion of YkuD domains comprising the conserved catalytic motif. The conserved motifs comprising the catalytic Cys nucleophile is highlighted in yellow. The consensus for this motif is SxGChR/A, where x is any residue and h is a hydrophobic residue.

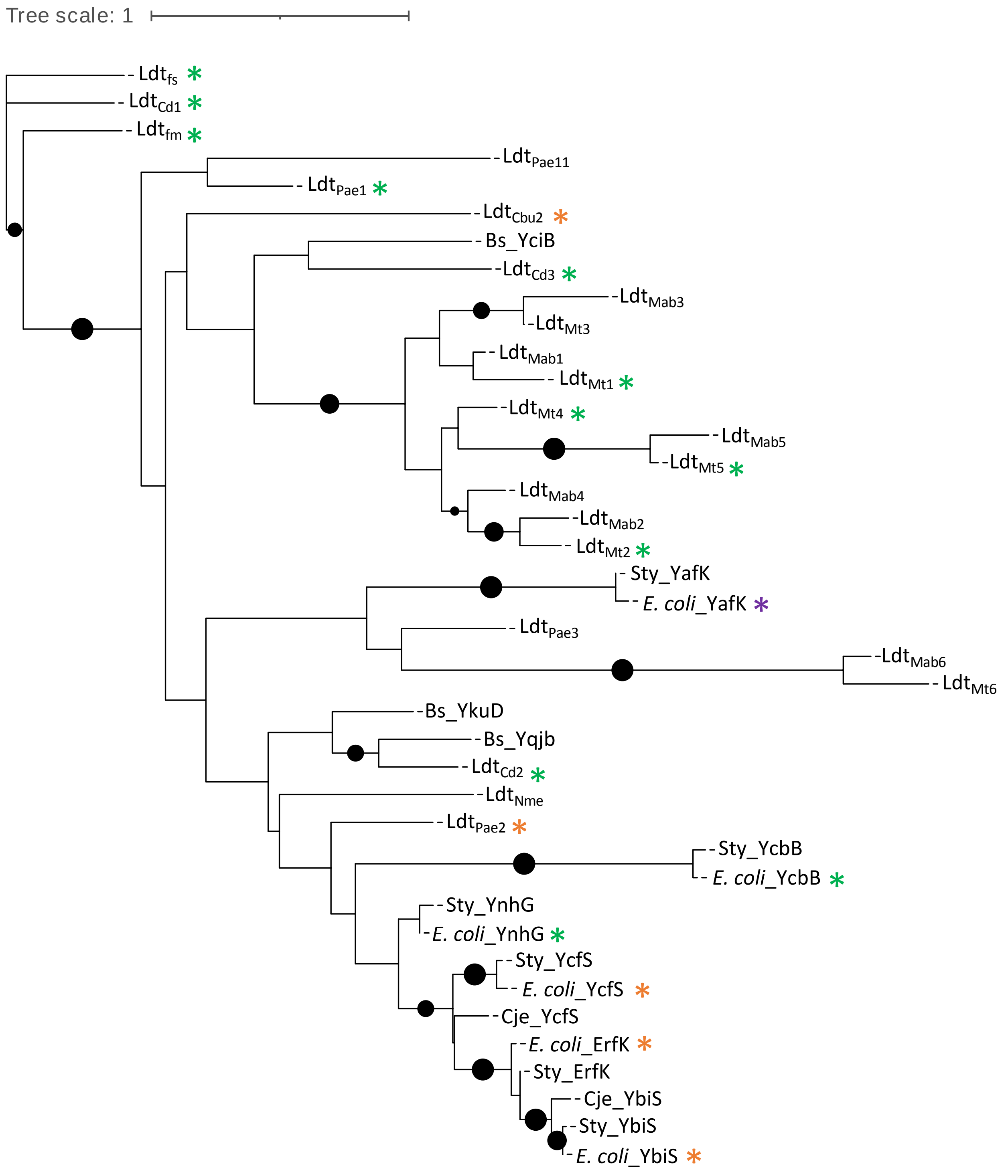

Supplementary Figure S3. Phylogram of the YkuD catalytic domains of L,D-transpeptidases. The phylogram was obtained on iTOL. Closed circles indicate bootstraps with a score greater than 95 and their size is proportional to the score. We consider that a score lower than 95 does not allow to affirm that the node between the two branches is supported. Stars indicate the function if known: green, 3→3 cross-linking; orange, lipoprotein or β-barrel protein anchoring; purple, hydrolysis of the tripeptide→lipoprotein amide bond.

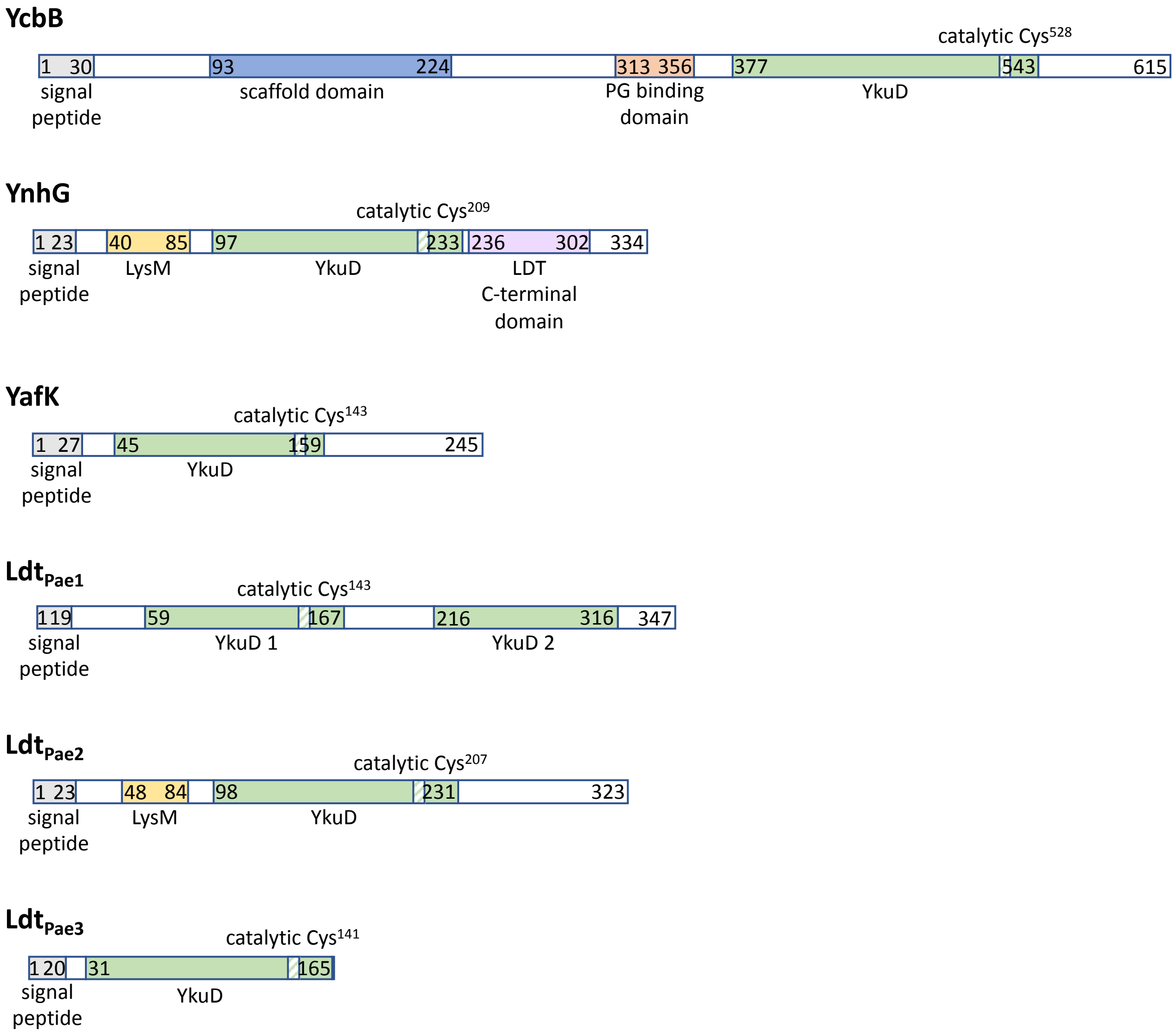

Supplementary Fig. S4. Domain architecture of relevant LDTs. Data were recovered from the InterPro data base.
